# Invasive fungal infection among hematopoietic stem cell transplantation patients with mechanical ventilation in the intensive care unit

**DOI:** 10.1186/1471-2334-12-44

**Published:** 2012-02-18

**Authors:** Chen-Yiu Hung, Kuo-Chin Kao, Po-Nan Wang, Han-Chung Hu, Meng-Jer Hsieh, Jui-Ying Fu, Chih-Hao Chang, Li-Fu Li, Chung-Chi Huang, Ying-Huang Tsai, Cheng-Ta Yang

**Affiliations:** 1 Department of Thoracic Medicine, Chang Gung Memorial Hospital, Chang Gung University College of Medicine, Taipei, Taiwan; 2 Department of Respiratory Therapy, Chang Gung Memorial Hospital, Chang Gung University College of Medicine, Taipei, Taiwan; 3 Department of Respiratory Care, Chang-Gung University College of Medicine, Taipei, Taiwan; 4 Division of Hematology-Oncology, Chang Gung Memorial Hospital, Chang Gung University College of Medicine, Taipei, Taiwan

**Keywords:** Invasive fungal infection (IFI), Hematopoietic stem cell transplantation (HSCT), Intensive care unit (ICU), Outcome assessment, Risk factor

## Abstract

**Background:**

Invasive fungal infection (IFI) is associated with high morbidity and high mortality in hematopoietic stem cell transplantation (HSCT) patientsThe purpose of this study was to assess the characteristics and outcomes of HSCT patients with IFIs who are undergoing MV at a single institution in Taiwan.

**Methods:**

We performed an observational retrospective analysis of IFIs in HSCT patients undergoing mechanical ventilation (MV) in an intensive care unit (ICU) from the year 2000 to 2009. The characteristics of these HSCT patients and risk factors related to IFIs were evaluated. The status of discharge, length of ICU stay, date of death and cause of death were also recorded.

**Results:**

There were 326 HSCT patients at the Linkou Chang-Gung Memorial Hospital (Taipei, Taiwan) during the study period. Sixty of these patients (18%) were transferred to the ICU and placed on mechanical ventilators. A total of 20 of these 60 patients (33%) had IFIs. Multivariate analysis indicated that independent risk factors for IFI were admission to an ICU more than 40 days after HSCT, graft versus host disease (GVHD), and high dose corticosteroid (*p *< 0.01 for all). The overall ICU mortality rate was 88% (53 of 60 patients), and was not significantly different for patients with IFIs (85%) and those without IFIs (90%, *p *= 0.676).

**Conclusion:**

There was a high incidence of IFIs in HSCT patients requiring MV in the ICU in our study cohort. The independent risk factors for IFI are ICU admission more than 40 days after HSCT, GVHD, and use of high-dose corticosteroid.

## Background

Hematopoietic stem cell transplantation (HSCT) is a treatment option for malignant and non-malignant hematological disorders such as leukemia, lymphoma, myelodysplastic syndrome, and aplastic anemia. Despite improvements in HSCT, its widespread adoption has been limited due to the high rate of severe complications associated with the toxicity of the conditioning regimen, immunosuppression, and graft-versus-host disease (GVHD) [1]. These complications are commonly related to the underlying illness and often require admission to an intensive care unit (ICU) [2,3]. More than 60% of HSCT recipients are admitted to an ICU, and these patients have very poor prognosis [4,5]. Although the mortality rate of HSCT recipients admitted to the ICU has declined in the past two decades, it still exceeds 80% for patients on mechanical ventilation (MV) [5].

Invasive fungal infections (IFIs) are increasingly diagnosed in critically ill HSCT patients. The main risk factors for an IFI are advanced age, use of an unmatched donor, neutropenia, acute GVHD, underlying disease, corticosteroid therapy, and duration of fungemia [6]. Other risk factors for serious fungal infection are the nature of the critical illness, use of immunosuppressive therapy, presence of neutropenia, use of multiple broad-spectrum antibiotics, use of total parenteral nutrition, and presence of an indwelling catheter [6]. Despite advances in HSCT procedures, improved prophylactic antifungal agents, and better ICU care, mortality and morbidity remain major concerns for HSCT patients with IFIs [7].

HSCT recipients with respiratory failure who need MV have increased risk of transplant-related morbidity and mortality, but few reports have examined the association of IFI and in these patients. The purpose of this study was to assess the characteristics and outcomes of HSCT patients with IFIs who are undergoing MV at a single institution in Taiwan.

## Methods

### Patient information and data collection

A total of 326 adult patients underwent HSCT in Linkou Chang-Gung Memorial Hospital (Taipei, Taiwan) between January 2000 and December 2009. Four patients whose families declined ICU admission and two patients who died before ICU admission were excluded. For patients with multiple ICU admissions during the study period, only the first admission episode was analyzed. Ultimately, a total of 60 HSCT patients who were undergoing mechanical ventilation (MV) and admitted to the medical ICU during the study period were enrolled. The Institutional Review Board and the Chang Gung Medical Foundation (IRB no. 99-2134B) approved this study. Consent was waived due to the retrospective nature of the study.

The medical records of 60 patients were retrospectively reviewed. On the first day of ICU admission, we recorded patient age, gender, underlying disease, date and type of transplantation (autologous or allogeneic), stem cell source (bone marrow or peripheral blood stem cells), laboratory analyses, hemodynamic status, renal and hepatic function, episodes of concurrent GVHD, cytomegalovirus (CMV) infection, and corticosteroid use. The acute physiology and chronic health evaluation (APACHE) II score and the sequential organ failure assessment (SOFA) [8] score were calculated for each patient within the first 24 h of ICU admission.

For patients with IFIs, we recorded the clinical characteristics, cytological or histological results, mean duration of diagnosis after ICU admission, and treatment. We also evaluated the stem cell source, donor type, presence of acute or chronic GVHD, CMV infection, neutrophil count, bacteremia, and corticosteroid use in all patients. The status of discharge, length of ICU stay, date of death, and cause of death were also recorded.

### Definitions

Each IFI patient was classified as having "proven IFI", "probable IFI", or "possible IFI" based on criteria of the European Organization for Research and Treatment of Cancer/Mycoses Study Group (EORTC/MSG) [9]. In brief, proven IFI was diagnosed by serologic analysis, microscopic analysis, and culture; probable IFI was diagnosed by host, clinical criteria, and mycological criteria; and possible IFI was diagnosed by the same criteria as probable IFI, except that mycological criteria were not used. The day on which the second positive diagnostic test was performed was defined as the day of IFI diagnosis. When an IFI was diagnosed by surgical lung biopsy or trans-bronchial lung biopsy, the day of diagnosis was defined as the day of the first clinical suspicion of IFI. IFI was defined as an early infection if it occurred within 40 days after HSCT, and was otherwise defined as a late infection. Previous studies have used this cutoff date as an indication of the immediate post-engraftment period [10].

Acute GVHD was defined as GVHD that occurred within 100 days after myeloablative chemotherapy [11]. The severity of acute GVHD in the three main target organs (skin, liver, gastrointestinal tract) was assigned stage 1 to 4 based on accepted criteria [12]. Chronic GVHD is determined by a 0- to 3-point score (none, mild, moderate, severe) that reflects the clinical effect in various organs including skin, liver, eyes, mouth, respiratory tract, and esophagus [13].

Cytomegalovirus (CMV) infection was diagnosed by positive culture and characteristic pathological findings in specimens from a surgical lung biopsy or bronchoalveolar lavage. Neutropenia was diagnosed as a neutrophil count less than 500 cells/mL for more than 10 days. Concurrent bacteremia was diagnosed if bacteria were isolated from blood culture within one week of the diagnosis of IFI. High dose corticosteroid use was defined as more than 0.3 mg/kg/day prednisolone for more than three weeks.

### Statistical analysis

Data were analyzed with SPSS 13.0 (SPSS Inc. Chicago, II, USA). The characteristics of with and without IFIs were compared by a Chi-square test for categorical variables and by an independent *t*-test for continuous variables. If the cells of a 2 × 2 table had an expected count less than five, Fisher's Exact Test was used. Risk factors for IFI were initially assessed by univariate analysis. Variables that were statistically significant (*p *< 0.05) in the univariate analysis were included in a multivariate analysis by a multiple logistic regression based on the backward elimination of data. All statistical tests were two-tailed and a *p*-value less than 0.05 was considered statistically significant.

## Results

A total of 326 patients underwent HSCT at our institution during the study period, and 66 of these patients had respiratory failure before ICU admission that necessitated MV. Six of those patients were excluded because they declined ICU admission or died before admission. Table 1 shows the baseline characteristics of the 60 enrolled patients.

**Table 1:** Characteristics of HSCT patients with mechanical ventilation (n = 60)

	Mean ± S.D. or Number (%)^a^
**Age at transplantation, yrs**.	34.5 ± 10.3

**Gender**	

Male	37 (61.6%)

Female	23 (38.4%)

**Underlying disease**	

ALL	9 (15%)

AML	14 (23.3%)

Aplastic anemia	2 (3.3%)

CML	12 (20%)

Multiple myeloma	4 (6.7%)

MDS	2 (3.3%)

Hodgkin's disease	4 (6.7%)

Non Hodgkin's lymphoma	8 (13.3%)

Other	5 (8.3%)

**Type of transplantation**	

Allo-PBSCT	36 (60%)

Auto-PBSCT	13 (21.7%)

Allo-BMT	10 (16.7%)

Auto-BMT	1 (1.7%)

**GVHD**	

Yes	31

No	15

**APACHE II score**	24.5 ± 9.4

**APACHE II > 25 (Patient number)**	27

**SOFA score**	12 ± 3.7

**Duration from HSCT to ICU, days**	106.4 ± 99.5

^a^Data represent the mean ± S.D or the number of patients and percentage in parentheses.

Abbreviations: HSCT: hematopoietic stem cell transplantation; ALL: acute lymphoid leukemia; AML: acute myeloid leukemia; CML: chronic myeloid leukemia; MDS: myelodysplastic syndrome; PBSCT: peripheral blood stem cell transplantation; BMT: bone marrow transplantation; GVHD: graft versus host disease; APACHE II: acute physiology and chronic health evaluation II; SOFA: sequential organ failure assessment.

A total of 20 of these 60 HSCT patients undergoing MV had IFIs (33%). Table 2 shows the basic characteristics of patients with and without IFIs. Five of the 20 patients diagnosed with IFIs were diagnosed within 30 days after HSCT, four patients were diagnosed between 30 and 100 days after HSCT, and 11 patients were diagnosed more than 100 days after HSCT. Patients with IFIs were significantly younger than those without IFIs (30 *vs*. 37 yrs., *p *= 0.024). IFI patients were significantly more likely to be diagnosed with GVHD and to have received high dose corticosteroid therapy (94% *vs*. 52%, *p *= 0.003 and 65% *vs*. 25%, *p *= 0.003, respectively). In addition, the APACHE II and SOFA scores were significantly lower in patients with IFIs (21.6 *vs*. 26.7, *p *= 0.014 and 10.5 *vs*. 12.8, *p *= 0.02, respectively). The overall ICU mortality rate was 88% (53/60), and was not significantly different between the two groups (85% *vs*. 90%).

**Table 2:** Characteristics of HSCT patients with and without invasive fungal infections

	Invasive fungal infection (n = 20)	Without invasive fungal infection (n = 40)	P-value	
**Median age at ICU admission**	30 ± 7.9	37 ± 11.0	0.024	*

**Gender**				

Male	12 (60%)	15 (37.5%)	0.099	

Female	8 (40%)	25 (62.5%)		

**Early vs. late ICU admission after HSCT (day)**	209 ± 158.2	73 ± 56.6	0.068	

**ICU length of stay (day)**	10.7 ± 7.6	12.8 ± 14.5	0.533	

**Underlying disease**				

ALL	4 (20%)	5 (12.5%)		

AML	8 (40%)	7 (17.5%)		

CML	3 (15%)	9 (22.5%)		

Multiple myeloma	1 (5%)	3 (7.5%)		

Hodgkin's disease	2 (10%)	2 (5%)		

Other hematological disease	2 (10%)	14 (34.5%)		

**Type of transplantation**				

Allo-PBSCT/Allo-BMT	13 (65%)/4 (20%)	24 (60%)/5 (12.5%)		

Auto-PBSCT/Auto-BMT	3 (15%)/0	10 (25%)/1 (2.5%)		

**GVHD**	16 (80%)	15 (37.5%)	0.003	**

**CMV infection**	7 (35%)	6 (15%)	0.101	

**Neutropenia**	16(80%)	32 (80%)	0.099	

**Concurrent bacteremia**	12 (60%)	26 (65%)	0.705	

**High dose corticosteroid**	13 (65%)	10 (25%)	0.003	*

**APACHE II score**	22 ± 5.7	27 ± 9.8	0.014	*

**SOFA score**	10.45 ± 2.7	13 ± 4.0	0.020	*

**ICU mortality**	17 (85%)	36 (90%)	0.676	

*: *p *< 0.05; **: *p *< 0.01.

Data represent as mean ± S.D or number (percentage).

Abbreviations: HSCT: hematopoietic stem cell transplantation; ALL: acute lymphoid leukemia; AML: acute myeloid leukemia; CML: chronic myeloid leukemia; MDS: myelodysplastic syndrome; PBSCT: peripheral blood stem cell transplantation; BMT: bone marrow transplantation; GVHD: graft versus host disease; APACHE II: acute physiology and chronic health evaluation II; SOFA score: sequential organ failure assessment score; and ICU: intensive care unit.

Table 3 shows the results of our multivariate analysis of variables associated with IFIs. The results indicate that ICU admission more than 40 days after HSCT, diagnosis of GVHD, and use of high dose corticosteroid were significantly and independently associated with IFIs.

**Table 3:** Univariate and multivariate analysis of clinical variables associated with invasive fungal infection

	Univariate analysis						Multi-variate analysis					
**Variable**	**Odds Ratio**	**95% C.I**.			**P-value**		**Odds ratio**	**95% C.I**.			**P-value**	

Age > 30	0.333	0.108	-	1.034	0.053							

Gender	0.900	0.300	-	2.704	0.099							

Late ICU admission (> 40 days after HSCT)	6.500	1.916	-	22.052	0.002	**	1.003	1.001	-	1.006	0.008	*

Type of transplantation	0.465	0.114	-	1.906	0.281							

GVHD	6.667	1.874	-	23.714	0.002	*	8.291	1.687	-	40.746	0.009	*

CMV infection	3.051	0.862	-	10.799	0.101							

Neutropenia (ICU admission)	1.000	0.261	-	3.826	0.099							

Concurrent bacteremia	0.808	0.267	-	2.440	0.705							

High dose corticosteroid use	5.571	1.738	-	17.856	0.003	*	9.459	2.040	-	43.867	0.004	*

APACHE II > 25	0.130	0.033	-	0.518	0.002	*						

SOFA > 12	0.250	0.071	-	0.880	0.025	*						

ICU Mortality	0.810	0.173	-	3.792	0.676							

a. If cells had expected count less than 5, Fisher's Exact Test was used.

b. *: *p *< 0.05; **: *p *< 0.01

Abbreviations: C.I.: confidence interval; HSCT: hematopoietic stem cell transplantation; GVHD: graft versus host disease; CMV: cytomegalovirus; APACHE II: acute physiology and chronic health evaluation II; SOFA score: sequential organ failure assessment score; ICU: intensive care unit.

**Figure 1:**
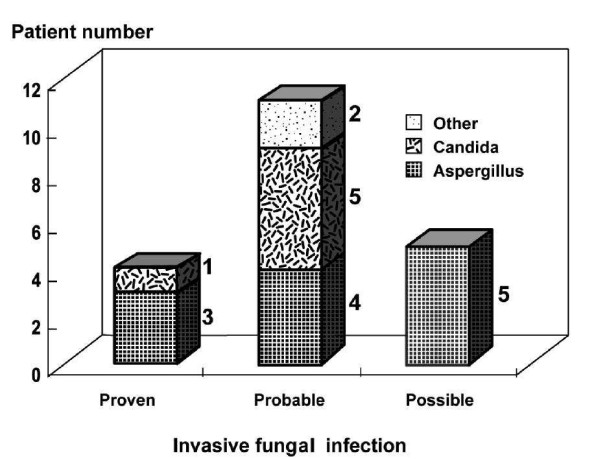
**Diagnostic categories and characteristics of patients with invasive fungal infections**.

The average time of IFI diagnosis was four days after ICU admission and the range was one to seven days. Figure 1 shows the diagnostic categories and characteristics of the 20 IFI patients. Four patients had proven IFIs, and 3 of these patients had *Aspergillus *and 1 patient had *Candida*. Eleven patients had probable IFIs, and 4 of these patients had *Aspergillus*, 5 patients had *Candida*, and 2 patients had other fungi. Five patients had possible IFIs, and all 5 patients had *Aspergillus*.

All 20 patients with IFIs had lung involvement, and two patients had concurrent central nervous system infection by *Aspergillus*. All 60 of the HSCT patients received empiric treatment with fluconazole if neutropenia was diagnosed. *Aspergillus *spp. was the most common isolate (12 of 20 patients). All patients with proven IFI and possible IFI died. Only three of the 20 patients with probable IFI survived, one with probable *Aspergillus *who was treated with amphotericin B, and two with probable *Candida *who were treated with fluconazole. For the three IFI patients who survived, one had *Aspergillus flavus *and the other two had *Candida *spp. other than *C. tropicalis *or *C. albicans*. Among the 17 IFI patients who died, the causes of death were multiple organ failure (12 patients), septic shock (3 patients), and profound hypoxemia (2 patients).

## Discussion

The major findings of this study were that HSCT patients undergoing MV in the ICU had a high incidence of IFI (33%) and high ICU mortality (88%). Patients with IFIs had a significantly longer duration from HSCT to ICU admission, higher percentage of GVHD, and greater use of high-dose corticosteroid use than patients without IFIs. Although univariate analysis indicated that IFI patients had lower APACHE II and SOFA scores, multivariate analysis indicated that these were not independently associated with IFI. Moreover, the ICU mortality rate was similar for patients with and without IFIs.

Several previous studies have reported high mortality rates in HSCT recipients who were admitted to ICUs. In particular, Soubani reported that intubated HSCT patients had an ICU mortality rate of nearly 100% before 1990, but that the mortality rate decreased to 61-67% in more recent years [6]. The mortality rate of these patients was directly related to the number of organ system failures and use of MV rather than their status as HSCT recipients [4,6]. Other research reported that early initiation of noninvasive positive ventilation decreased the likelihood of invasive endotracheal intubation, and can improve the survival rate of HSCT patients with respiratory failure [5]. The high ICU mortality rate in the present study (88%) may be partially explained by the fact that all enrolled patients were receiving invasive MV upon ICU admission.

Previous reports indicated that impaired cell-mediated immunity and use of immunosuppressants to treat GVHD led to CMV reactivation in HSCT patients [14,15]. In particular, CMV reactivation was identified as a significant independent risk factor for HSCT patients with IFIs during the early post-engraftment phase [14,15]. The HSCT patients undergoing MV with IFIs in the present study also had higher percentage of CMV infection than those without IFIs (7 of 20, 35% *vs*. 6 of 40, 15%, respectively), although this difference was not statistically significant.

Previous studies reported that the risk factors for IFI in HSCT recipients were presence of an underlying disease, advanced age, donor type, GVHD, and use of corticosteroids [16-18]. Moreover, higher grade GVHD and higher dose of corticosteroid were associated with increased likelihood of an IFI [19,20]. Other studies reported that neutropenia and use of immunosuppressants to treat GVHD were associated with IFI [21,22]. The presence of neutropenia increases the risk of developing bacterial or fungal infections. During the neutropenic period, HSCT patients have increased risk of developing severe sepsis, septic shock, and multiple organ failure, making it necessary for more intensive care that includes MV. However, the present study of HSCT patients undergoing MV indicated that significant risk factors for IFI were GVHD and use of high-dose corticosteroid, but not neutropenia. The very high overall rate of neutropenia in our severely ill patients (80%) may partially explain why there was no significant difference in neutropenia between patients with and without IFIs.

During our study period, 7 of 326 HSCT patients were admitted to the ICU without MV. Among these 7 non-ventilated critically ill patients, 4 patients had IFIs (57%) and all 4 of these patients were diagnosed with possible IFI by *Aspergillus *spp. following ICU admission more than 40 days after HSCT. Two of these 4 patients died. Clearly, a larger number of patients are needed to establish the influence of IFI on the outcome of non-ventilated critically ill HSCT patients. A previous study reported that IFIs in HSCT patients differed according to the time of infection after HSCT [14]. IFI cases that occurred within 40 days of HSCT were predominantly *Candida *infections and were more common in patients with active hematological disease, longer duration of neutropenia, and severe GVHD [6,14]. IFI cases that occurred more than 40 days after HSCT were predominantly *Aspergillus *infections, and were more common in patients with mismatched related donor transplants, severe GVHD, and use of high-dose corticosteroid [6,14]. In the present study, IFI was diagnosed a mean of 209 days after HSCT, during the late post-engraftment phase. The use of a non-myeloablative conditioning regimen and granulocyte-colony stimulation factor (G-CSF) may improve the transplantation success and reduce the duration of neutropenia. Furthermore, these improvements appeared to decrease the incidence of early IFI, but increase the incidence of late IFI.

The GITMO (Gruppo Italiano Trapianto di Midollo Osseo) study reported that Aspergillosis accounted for 80% of invasive fungal infections in HSCT patients [23]. In our patients with IFIs, 60% (12 of 20 patients) had Aspergillosis, and *Aspergillus flavus *was the most common species (3 of 11 patients). In the early 1990s, *Candida albicans *accounted for about two-thirds of *Candida *infections, but this decreased to about 50% by the end of the last decade [24]. Our results indicated that *Candida albicans *was the most isolated species of *Candida *(50%, 3 of 6 patients).

HSCT patients who have IFIs have poor prognosis, and the overall mortality rate of our HSCT patients undergoing MV was 88%. Our results also indicated similar ICU mortality rates in patients with and without IFIs. Thus, the presence of an IFI had no further impact on the already dismal prognosis of HSCT patients undergoing MV.

There were three major limitations in our study. First, we included patients who were diagnosed with "possible IFI" because of the difficulty in establishing a proven or probable diagnosis. It is possible that some of these "possible IFI" patients did not actually have IFIs. If so, then the differences between the two groups may have been reduced. Second, our study was retrospective and was performed at a single tertiary care medical center, so the findings have limited generalizability. Third, antifungal azoles and echinocandins have only been available in the last five years at our institute, so other populations with longer histories of azole and echinocandin use may have different results.

## Conclusion

HSCT patients undergoing MV in the ICU had a high rate of IFI and had an extremely high rate of mortality. The risk factors for IFI in HSCT patients undergoing MV were long time from HCST to ICU admission, presence of GVHD, and use of high-dose corticosteroid. Recognition of risk factors for an IFI and early use of effective antifungal agents that can treat *Aspergillus *and fluconazole-resistant *Candida *spp. may provide better outcomes for HSCT patients with IFIs.

## Key message

• Hematopoietic stem cell transplantation (HSCT) patients undergoing mechanical ventilation (MV) in the intensive care unit (ICU) had a high rate of invasive fungal infection (IFI) and an extremely high rate of mortality.

• The risk factors for IFI in HSCT patients undergoing MV were late ICU admission after HSCT, GVHD, and use of high dose corticosteroid.

• In our study cohort, *Aspergillus *was the primary cause of IFI in and infection mostly occurred during the late post-engraftment phase.

## Abbreviations

ALL: Acute Lymphoid Leukemia; AML: Acute Myeloid Leukemia; APACHE II: Acute Physiology and Chronic Health Evaluation II; BMT: Bone Marrow Transplantation; CML: Chronic Myeloid Leukemia; CMV: Cytomegalovirus; EORTC/MSG: European Organization for Research and Treatment of Cancer/Mycoses Study Group; G-CSF: Granulocyte-Colony Stimulation Factor; GITMO: GRUPPO Italiano Trapianto di Midollo Osseo; GVHD: Graft-Versus-Host Disease; HSCT: Hematopoietic Stem Cell transplantation; IFI: Invasive Fungal Infection; ICU: Intensive Care Unit; MDS: Myelodysplastic Syndrome; MV: Mechanical Ventilation; PBSCT: Peripheral Blood Stem Cell transplantation; SOFA: Sequential Organ Failure Assessment.

## Competing interests

The authors declare that they have no competing interests.

## Authors' contributions

CYH and KCK conceived and designed the study and wrote the article. PNW recruited patients. The study was performed by HCH, CCH, MJH, CHC, JYF, LFL, and YHT. The data was coordinated and analyzed by CTY. All the authors contributed to, read, and approved the final manuscript.

## Pre-publication history

The pre-publication history for this paper can be accessed here:

http://www.biomedcentral.com/1471-2334/12/44/prepub
